# Multi-Omics Approach Identifies Molecular Mechanisms of Plant-Fungus Mycorrhizal Interaction

**DOI:** 10.3389/fpls.2015.01061

**Published:** 2016-01-19

**Authors:** Peter E. Larsen, Avinash Sreedasyam, Geetika Trivedi, Shalaka Desai, Yang Dai, Leland J. Cseke, Frank R. Collart

**Affiliations:** ^1^Argonne National Laboratory, Biosciences DivisionLemont, IL, USA; ^2^Department of Bioengineering, University of Illinois at ChicagoChicago IL, USA; ^3^Department of Biological Sciences, University of Alabama in HuntsvilleHuntsville, AL, USA

**Keywords:** *Laccaria bicolor*, *Populus tremuloides*, mycorrhizae, metabolomics, transcriptomics, proteomics, system modeling

## Abstract

In mycorrhizal symbiosis, plant roots form close, mutually beneficial interactions with soil fungi. Before this mycorrhizal interaction can be established however, plant roots must be capable of detecting potential beneficial fungal partners and initiating the gene expression patterns necessary to begin symbiosis. To predict a plant root—mycorrhizal fungi sensor systems, we analyzed *in vitro* experiments of *Populus tremuloides* (aspen tree) and *Laccaria bicolor* (mycorrhizal fungi) interaction and leveraged over 200 previously published transcriptomic experimental data sets, 159 experimentally validated plant transcription factor binding motifs, and more than 120-thousand experimentally validated protein-protein interactions to generate models of pre-mycorrhizal sensor systems in aspen root. These sensor mechanisms link extracellular signaling molecules with gene regulation through a network comprised of membrane receptors, signal cascade proteins, transcription factors, and transcription factor biding DNA motifs. Modeling predicted four pre-mycorrhizal sensor complexes in aspen that interact with 15 transcription factors to regulate the expression of 1184 genes in response to extracellular signals synthesized by Laccaria. Predicted extracellular signaling molecules include common signaling molecules such as phenylpropanoids, salicylate, and jasmonic acid. This multi-omic computational modeling approach for predicting the complex sensory networks yielded specific, testable biological hypotheses for mycorrhizal interaction signaling compounds, sensor complexes, and mechanisms of gene regulation.

## Introduction

Terrestrial plants process about 15% of total atmospheric carbon dioxide each year, drawing about 450 billion tons of carbon dioxide from the atmosphere (Beer et al., 2010). Depending on conditions and on ecosystems, between 20% (Gamper et al., 2005) and as much as 40% (Drigo et al., 2010) of that fixed atmospheric carbon is incorporated directly by subsurface mycorrhizal fungi living in symbiosis with plant roots. From this, it can be estimated that mycorrhizal symbiosis is a terrestrial sink of around 100 billion tons of carbon dioxide annually. In exchange for this carbon, symbiotic fungi provide access to nutrients otherwise inaccessible to the plant and protect the roots from a variety of biotic and abiotic stresses (Smith, 1974; Smith et al., 2003; Pozo and Azcón-Aguilar, 2007; Nehls, 2008; Bonfante and Genre, 2010). Ectomycorrhizal interaction involves differentiation of specialized fungal tissues and substantial reorganization of the root structure (Tagu and Martin, 1995). These symbiotic relationships between plant root and soil fungus depend on the activity of specific sensor complexes that receive information about a plant root's environmental conditions and potential symbiotic partners and use that information to initiate the molecular machinery required to establish and maintain the mycorrhizal interaction. Mycorrhizal-associated sensor complexes are likely comprised of multiple components: transmembrane receptors that detect specific signaling molecules in the extracellular environment, signal cascades that conduct information to the extracellular environment into the nucleus, transcription factors whose activity is modified as a consequence of the signal, and sets of genes regulated by those transcription factors whose encoded proteins enable the cell to respond to the extracellular environment. The inherent complexity of this sensor system requires multiple forms of biological experimental datasets to illuminate the full scope of system interactions. To infer sensor complexes for establishing and maintaining mycorrhizal symbiosis, we have combined archived transcriptomic data, databases of high-throughput proteomic data, genomic sequence analysis and metabolomic modeling with deep RNA sequencing analysis of a laboratory *in vitro* system comprised of the tree *Populus tremuloides* (aspen) and the ectomycorrhizal fungus *Laccaria bicolor* (Laccaria).

Using Laccaria and aspen as model organisms for symbiotic interactions provides a unique opportunity to investigate the molecular mechanisms of mycorrhizal interaction. Aspen and Laccaria are capable of forming mycorrhizae in the laboratory, making them useful models of plant-fungus symbiotic interactions. The genomes for both Laccaria and the closely related aspen species *Populus trichocapra* have been sequenced through the efforts of the Joint Genome Institute (JGI) providing a set of annotated gene models for use in transcriptomic analysis (Tuskan et al., 2006; Martin and Selosse, 2008) (http://jgi.doe.gov). The nature of the symbiosis that forms between aspen roots and Laccaria is termed ectomycorrhizal (ECM) symbiosis. In ECM interactions, the plant roots and fungus remain separated by a narrow space called the apoplast, requiring that nutrients and signaling molecules exchanged by plant and fungus be transported across both fungal and plant cell walls by specific transmembrane transporters and receptors.

We used several computational analysis approaches that accommodate multiple “omics” data sources to predict aspen mycorrhizal sensors. Using transcriptomics and metabolomic modeling, we identified possible signal compounds synthesized by Laccaria during pre-mycorrhizal interaction. Using transcriptomic data and published databases of known plant protein-protein interactions, we constructed possible protein-protein interaction networks that link transmembrane transporters to transcription factors via networks of signal cascade proteins. These sensor complexes can be linked, using transcriptomics data, to clusters of co-regulated genes that share common regulatory motifs. Together, these predictions can be combined into a system-scale model of pre-mycorrhizal interaction that spans Laccaria signaling compounds to the sensors that detect them, to the pre-mycorrhizal specific gene expression patterns in aspen root. This predicted system-scale interaction network model proposes molecular biological experiments that can validate these predictions and demonstrate the molecular mechanisms that mediate pre-mycorrhizal interaction.

## Methods

**Figure 1:**
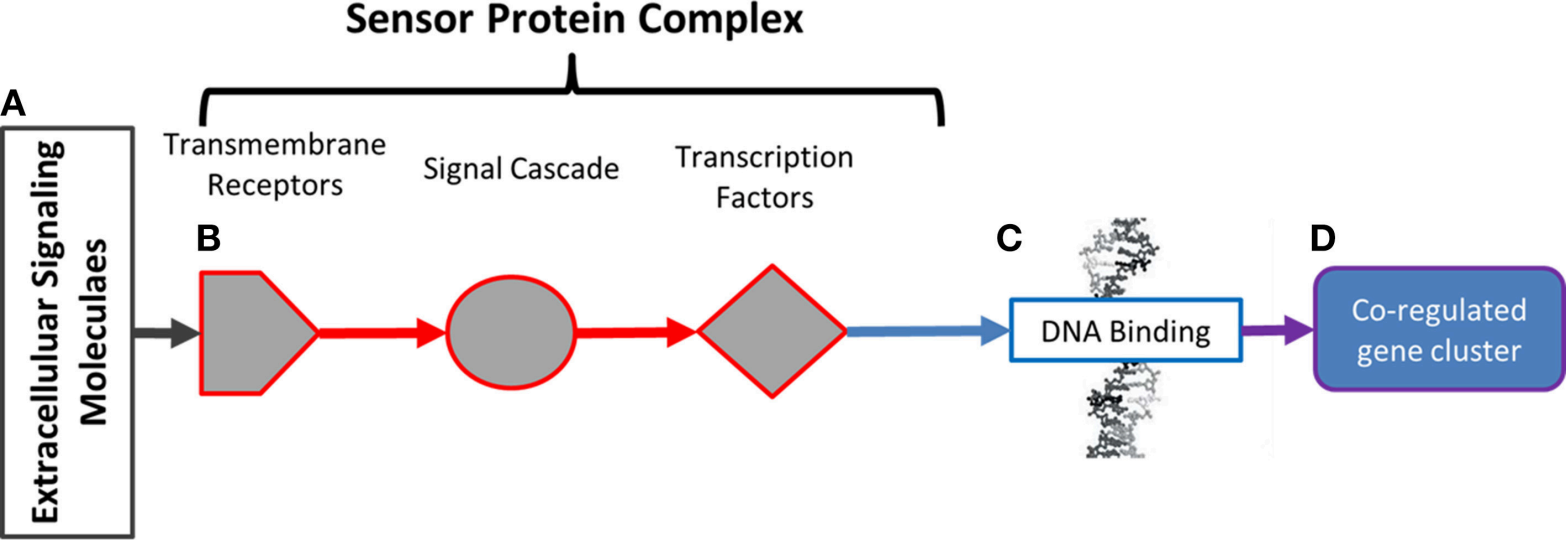
**Model of environmental sensor mechanisms**. In **(A)**, extracellular molecules contain information about extracellular parameters. That information is detected and conveyed to the nucleus by protein sensor mechanisms in **(B)**. These mechanisms are comprised of transmembrane receptors (hexagons) that bind to extracellular ligands. Information about extracellular conditions is relayed from the membrane receptors to the nucleus via signal cascade proteins (circles). Signal cascade regulates activities of transcription factors (diamonds). By binding to specific DNA motifs in the genome **(C)**, regulation of transcription factor activity drives patterns of co-regulated gene expression **(D)**. Patterns of gene expression drive observable phenotypic changes in the aspen root.

**Figure 2:**
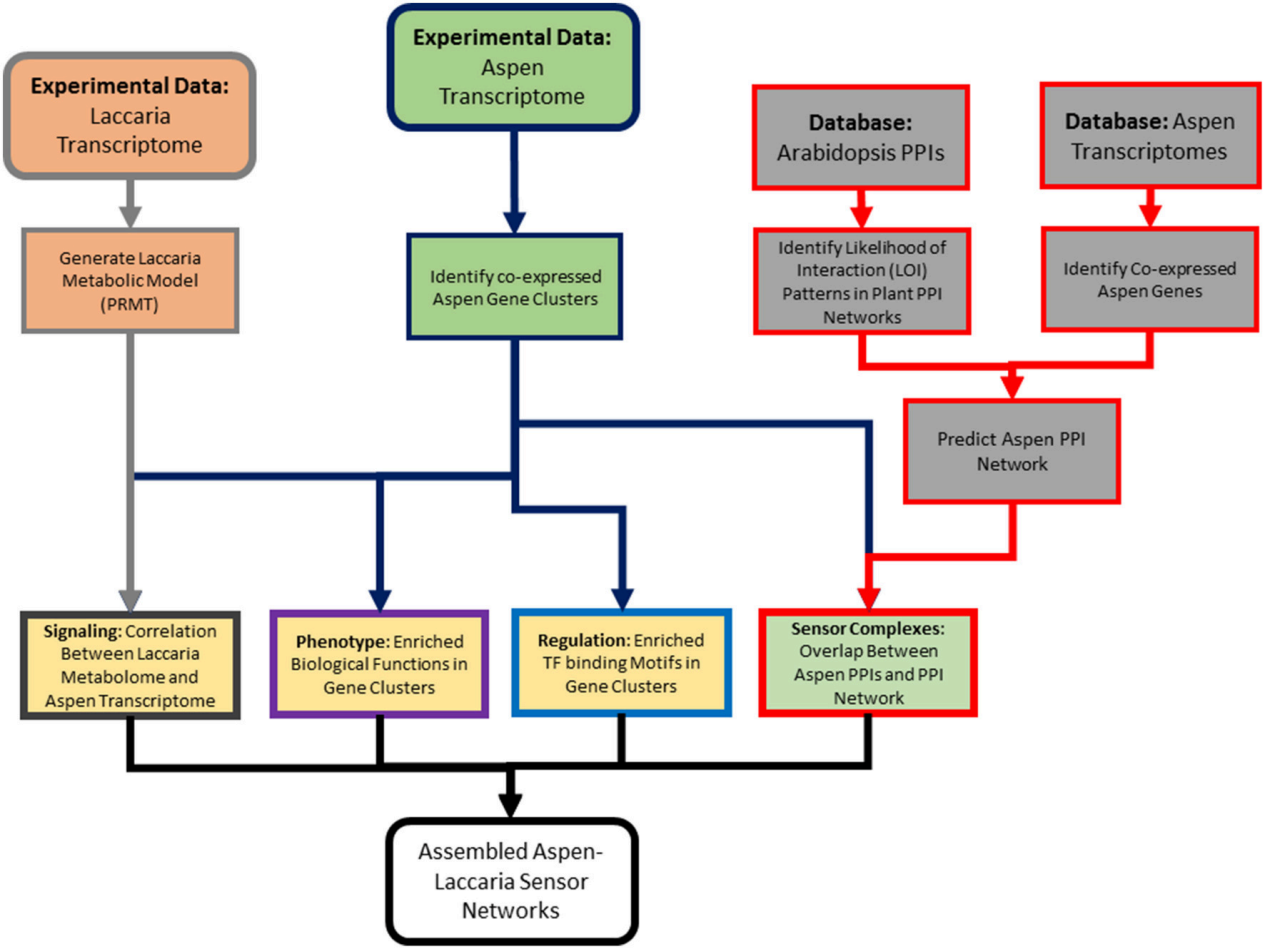
**Outline of analysis pipeline**. This high-level diagram outlines the multi-omics data used in analysis, what individual analysis methods were applied, and how those multi-omic data types were integrated. Box border colors reference the colors used for identifying components if sensor mechanisms from Figure 1.

The conceptual model of mycorrhizal sensor mechanisms used as a framework for subsequent data analysis is represented in Figure 1. In this model, extracellular ligands contain information about potential symbiotic interactions (Figure 1A). The ligands in this system are the metabolic products of Laccaria. Ligands are detected and that information is conveyed to the nucleus by protein sensor mechanisms (Figure 1B). These mechanisms are comprised of three components. Transmembrane receptors bind to extracellular ligands. Information about extracellular conditions is relayed from the membrane receptors to the nucleus via a network of signal cascade proteins. Finally, signal cascades regulate the activities of transcription factors. By binding to specific DNA motifs in the genome (Figure 1C), regulation of transcription factor activity drives patterns of co-regulated gene expression (Figure 1D). These patterns of gene expression drive phenotypic changes in the aspen root. An approach for generating this conceptual model from transcriptomic experimental and previously collected transcriptomic, genomic, and proteomic data is summarized in Figure 2.

### Laboratory biological system

The core component of this analysis is a laboratory experiment in which aspen and Laccaria are co-cultured in the laboratory. A crucial experimental aspect is the culturing of aspen and Laccaria together, but separated by a permeable membrane preventing direct contact between plant and fungus. In this condition, aspen and Laccaria can only interact via small signaling molecules. We hypothesize that observed changes in gene expression patterns in aspen root under these conditions, relative to aspen grown in monoculture, are potentially attributable to aspen root responding to diffusible signals generated by Laccaria. From laboratory cultures, RNA is extracted and sequenced. Sequenced mRNA is used to generate gene expression data for both Laccaria and aspen root. This transcriptomic data is analyzed in the context of prior transcriptomic and proteomic data sets, used to generate metabolomic models, and analyzed to identify significant genomic features associated with mycorrhizal interaction in later analysis steps.

#### Plant-fungal co-culture

**Figure 3:**
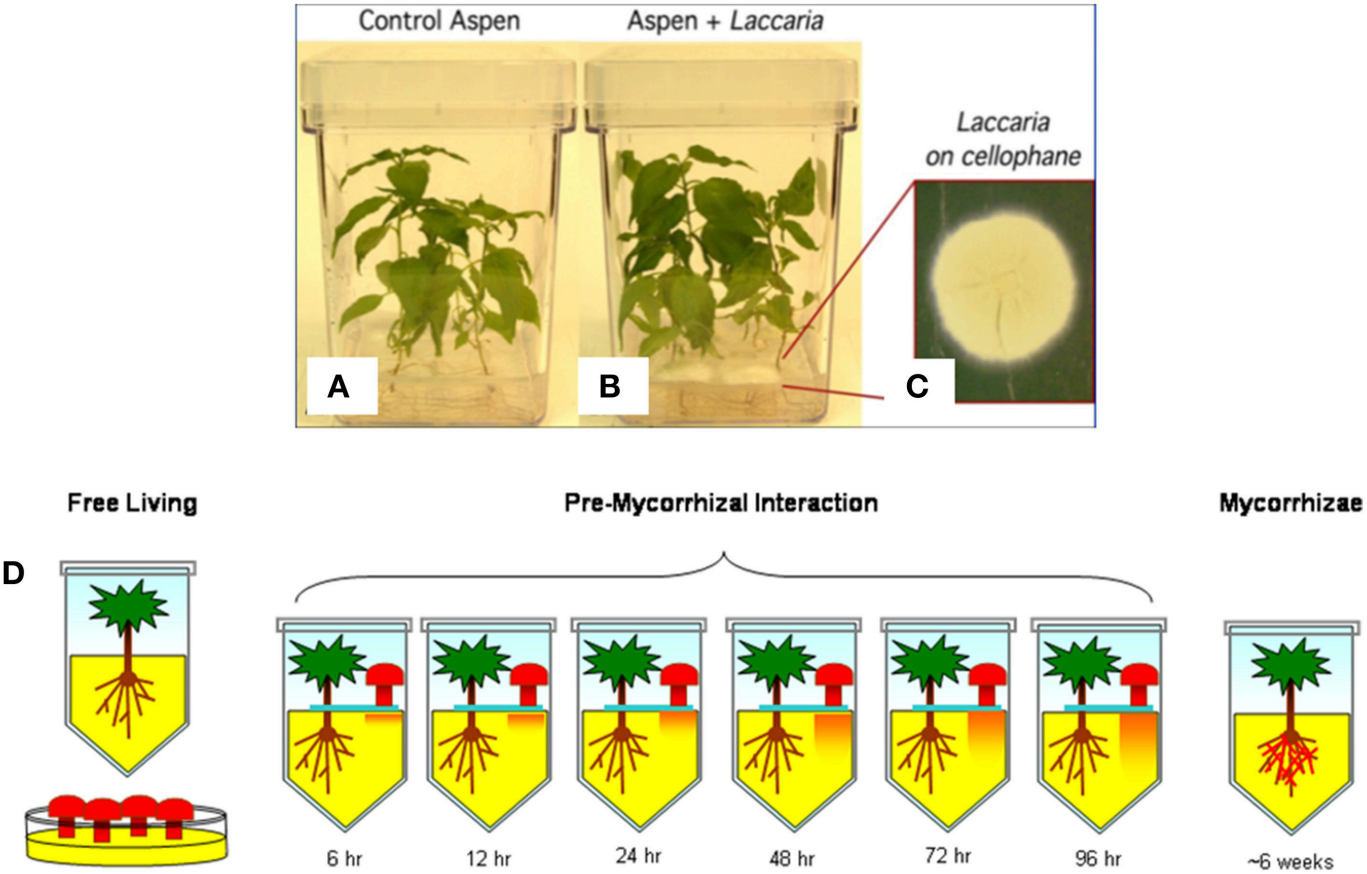
**Outline of biological system experimental design**. A typical example of laboratory aspen and Laccaria interaction cultures are pictured. **(A)** Aspen seedlings grown in monoculture. **(B)** Aspen and Laccaria grown in co-culture after 96 h. In this co-culture condition, Laccaria mycelia are cultured on a membrane **(C)** and transplanted onto surface of aspen seedling cultures, as in **(B)**. The separating membrane permits exchange of diffusible signaling molecules between aspen roots and Laccaria mycelia, but prevents physical contact between organisms. There were a total of nine experimental conditions that ranges from free-living aspen seedling and Laccaria mycelium, to co-cultured but physically separated by a permeable membrane to fully formed mycorrhizae **(D)**. In the cartoon, while Laccaria is symbolized by mushroom shapes, no fruiting bodies were actually present in experimental design.

Laboratory cultures of aspen and Laccaria were used to identify gene expression patterns specifically associated with mycorrhizal interaction via diffusion of small signaling molecules from Laccaria (Figure 3). There were three culture conditions considered: Free living, interaction, and mycorrhizal (Figure 3A). In free living states, aspen and Laccaria were cultured alone. In interaction, aspen and Laccaria were co-cultured, but separated by a membrane that permits diffusion of small molecules but does not allow direct physical contact between plant and fungus (Figure 3B). In mycorrhizal state, Laccaria was allowed to fully form mycorrhizal interactions with aspen roots (Figure 3C).

Laccaria (Maire) Orton (strain S238N) culture was maintained on Modified Melin Norkan's (MMN) media at 20°C, as described in Kim et al. (1998). *P. tremuloides* seeds were surface sterilized and germinated on McCown's woody plant media (WPM) in Petri dishes as previously described (Cseke, 2004). One week old germinated seedlings were transferred to Magenta vessels (Sigma, St. Louis, MO) containing the interaction medium (WPM with 1.5% of sucrose). The seedlings were grown under 16 h light and 8 h dark cycles at 24°C for 4–5 weeks until fine lateral roots were developed. Laccaria mycelial plugs were transferred to Magenta vessels on the surface of plant media or, for pre-mycorrhizal interaction samples, on a permeable cellophane membrane. Pre-mycorrhizal interaction aspen root tissue and Laccaria mycelia were collected, snap frozen in liquid nitrogen, and stored at −80°C at six time points: 6, 12, 24, 48, 72, and 96 h after initial contact. For fully formed mycorrhizae, aspen and Laccaria were co-cultured for ~6 weeks to form mycorrhizal interactions.

There were a total of 18 sequenced transcriptomes: 2 replicates of free living Laccaria, 2 replicates of free living aspen root, 6 interaction Laccaria (one sample per time point), 6 interaction aspen root (one sample per time point), and 2 replicates of fully-formed mycorrhizae. Fully formed mycorrhizae transcriptomes are comprised of comingled aspen and Laccaria transcriptomes. Of the 18 transcriptomes, 8 are Laccaria, 8 are aspen root, and 2 are mixed Laccaria and aspen root mycorrhizae.

#### RNA extraction and sequencing

Total RNA was extracted from samples by CTAB method (Cseke, 2004) and RNA quality was assessed by gel electrophoresis prior to library preparation. For mycorrhizal samples, extracted total RNA was comprised of both plant root and mycorrhizal fungus. Total RNA was treated with RQ-DNase (Promega, Madison, WI). Procedures described for preparation of mRNA for the mouse transcriptome analysis (Mortazavi et al., 2008) were used with some modifications. Ten micrograms of total RNA from each sample was hybridized to Sera-mag oligo (dT) beads (Thermo Scientific) for mRNA purification. Purified mRNA was fragmented by addition of 5X fragmentation buffer (Illumina, Hayward, CA) and was heated for 5 min at 94°C. First strand cDNA was synthesized using random primers to eliminate the general bias toward 3′ end of the transcript. Second strand cDNA synthesis was done by adding GEX second strand buffer (Illumina, Hayward, CA), dNTPs, RNaseH and DNA polymerase I followed by incubation for 2.5 h at 16°C. Second strand cDNA was further subjected to end repair, A-tailing, and adapter ligation in accordance with the manufacturer supplied protocols. Purified cDNA templates were enriched by PCR amplification with Phusion DNA polymerase (Illumina, Hayward, CA) and the samples were cleaned using QIAquick PCR purification columns and eluted in 30 μl EB buffer as per manufacturer's instructions (QIAGEN, CA). Purified cDNA libraries were quantified using Nanodrop spectrophotometer and loaded onto Illumina flow cells.

#### Determine gene expression from short read sequence data

Using a strategy we have previously employed (Larsen et al., 2010b, 2011b) and other investigators (Felten et al., 2009; Payyavula et al., 2009; Grisel et al., 2010), we used gene models from the JGI sequenced and closely related *P. trichocapra* as surrogates for *P. tremuloides* genes. Gene models and their annotations were taken from the DOE JGI website (www.jgi.doe.gov).

Gene model expression was detected in the collected transcriptomics data using the application “Bow**Strap**” (Larsen and Collart, 2012) (http://www.bio.anl.gov/molecular_and_systems_biology/BowStrap.html). “BowStrap” performs a bootstrap analysis on the output of the short sequence-aligning program “Bow**tie**” (http://bowtie-bio.sourceforge.net/index.shtml) In “BowStrap,” both unique and multiply aligned reads are considered to generate a measure of gene model expression with an accompanying confidence interval and statistical significance of expression. Bowtie indexes were generated from sets of published JGI gene models for *L. bicolor* and aspen For mycorrhizal samples, which contain sequence derived from both plant root and fungal transcriptomes, sequence reads were aligned to each set of indexed gene models. The default Bowtie conditions were used to generate alignments for all sets of sequence reads to gene models, except for setting Bowtie to return all possible sequence alignments. Ten thousand “BowStrap” iterations were used for the calculation of average and standard deviations of RPKM values. Significantly detected gene expression was defined as a Cumulative Normal Distribution (CND) based, uncorrected *p* < 0.0001.

### Analysis of aspen transcriptomic data

While transcriptomic data is collected from both the aspen and Laccaria components of the biological system, that data will be utilized very differently for aspen than for Laccaria. From aspen transcriptomes, we identified groups of co-regulated genes, found transcription factor binding sites that are enriched in those gene clusters, and predicted possible protein-protein interaction sensor complexes that potentially link the ability to detect extracellular conditions and use that information to drive changes in aspen root gene expression.

#### Co-regulated gene clusters in aspen root

We anticipate that groups of genes that are co-regulated in response to pre-mycorrhizal interaction conditions share relevant common functions for symbiotic interaction. For aspen root transcriptomes, gene models that were expressed in all experimental samples and were highly variable in expression across conditions were grouped into co-regulated sets of genes using K-means clustering. K-means clustering is a method by which multiple observations can be partitioned into a number of clusters in which each cluster shares similar means. For inclusion in clusters, genes were identified as significantly expressed in all 10 aspen root transcriptomes (2 × free living, 6 × interaction, and 2 × mycorrhizal transcriptomes) and differentially expressed as identified by a coefficient of variation >0.33 (Coefficient of variation is equal to the standard deviation of a measurement divided by the average measurement). K-means clustering was performed using Multiple Array Viewer (Saeed et al., 2003). The number of clusters was empirically determined from analysis of the data. A range of clusters sizes were tested and the resulting patterns of clustered gene expression are considered in the context of biological expectation: i.e., differentially expressed in mon-culture, differentially expressed in interaction, and differentially expressed in fully-formed mycorrhizae.

Statistically significantly (Cumulative Bionomical Distribution calculated *p* < 0.05) enriched Gene Ontology (GO) Biological Process annotations (Ashburner et al., 2000), relative to the distribution of annotations in genome, were identified in each gene cluster.

#### Identify transcription factor binding sites

Co-regulated genes in aspen root are expected to share common regulatory genomic elements and those elements are hypothesized to be associated with mechanisms of pre-mycorrhizal interaction. We combined transcriptomic analysis with genomic sequence analysis to identify possible transcription factor binding sites that are enriched in the identified co-regulated gene clusters.

Sequences 1000 bp upstream of co-regulated genes were searched for all instances of known DNA binding motifs. Possible transcription factor DNA binding sites were collected from PLAnt Cis-acting regulatory DNA Elements (PLACE, v30.0) (Higo et al., 1999), a publically available database of 469 experimentally validated plant transcription factor binding motifs. If at least one instance of a motif was observed upstream of a gene, then that motif was associated with that gene. While the number of times a motif is present in an upstream region is biologically relevant, only presence or absence of an upstream motif was considered for this analysis.

Two methods were used to identify putative mycorrhizal-associated transcription factor binding sites upstream of co-regulated aspen genes.

##### Enriched motifs in co-regulated gene clusters

Cumulative Binomial Distribution (CBD) was used to calculate statistical significance of enrichment for a transcription factor biding motif *M* in co-expressed gene cluster *C*:

$$\begin{array}{l} P\;-\;value\;=\;1-\overset{x_{C}^{M}}{\underset{y=0}{\sum }}\left(\begin{array}{c} n_{C}\\ y \end{array}\right){f_{M}}^{n_{C}}{\left(1-f_{M}\right)}^{\left(n_{C}-x_{C}^{M}\right)} \end{array}$$

where: *n*_*C*_ = number of genes in co-regulated gene cluster *C*.

$x_{C}^{M}$ = number of genes in co-regulated cluster *C* with motif *M* in 1000 bp upstream region.

*f*_*M*_ = frequency at which motif *M* is found in 1000 bp upstream region for all differentially regulated genes.

A significance threshold 0.01 and a requirement that at least 10 genes in a cluster to be associated with a particular motif were used to identify enriched motifs in co-regulated gene clusters. These values for significance and number of genes were empirically determined from the distribution of transcription factor binding motifs in this data. A moderate significance level of 0.01 was used due to the expectation that all genes in a co-regulated cluster are not likely driven by the same transcription factor. A minimum of 10 genes indicates that a potential motif regulates a cluster of genes actually differentially regulated in this experiment and not just co-occurring in genes that share biological function.

##### Transcription factors binding motifs in predicted sensor complex

Known transcription factor binding motifs for transcription factors present in predicted sensor complexes present in co-regulated gene clusters were identified. The upstream regions for co-regulated genes were searched for these motifs.

#### Predict sensor complexes in aspen root

Any signaling molecules synthesizes by Laccaria will have to be detected by membrane-bound sensors in aspen roots, then that signal relayed via signal cascade interactions to alter the activity of specific transcription factors. While no direct proteomic information was collected, the patterns of gene expression in roots in conjunction with databases of known plant protein-protein interactions will be used to identify possible sensor complexes that have the ability to link the synthesis of signaling molecules to resulting changes in aspen gene expression.

An integrated analysis of our *in vitro* transcriptomic data, genomic annotations, previously reported aspen root transcriptomes under a wide variety of growth conditions, and tens of thousands of previously validated plant protein-protein interactions was used to predict protein complexes in aspen root. We required the following conditions for inferring the existence of a Protein-Protein Interaction (PPI) involved in signal detection and gene regulation:

1. All proteins in a PPI must be significantly expressed at every time point.
2. Proteins in a PPI must be annotated with a function relevant to environmental sensing and signal transduction (List of annotations used us found in Table S1).
3. Proteins in a PPI complex must be co-expressed across a range of biological conditions.
4. The interaction network must follow a topology expected for biological networks.
5. Proteins in PPI networks must be predicted co-occur in sub cellular locations that are conducive to direct, physical interactions.
6. Predicted PPIs are considered to occur only between proteins within an organism and not between organisms.

To identify putative expressed sensor protein complexes, we used the following computational approaches. We presume that if a gene model is detected as significantly expressed, then its protein product is present as well. Transcriptomic data archived in the Gene Expression Omnibus (GEO) (http://www.ncbi.nlm.nih.gov/geo/) were used to identify sets of genes that are co-expressed under multiple biological conditions. One hundred and sixty three transcriptomes for *Populus* roots (GSE20118) and 14 tissue-specific transcriptomes (GSE21481) were collected for a total of 188 aspen transcriptomic data sets.

To generate co-expression networks that follow topology expected of biological networks (Chen et al., 2008; Larsen et al., 2010a), we used the rank-based method Gene annotation Restricted Value Neighborhood (GRV-N) (Larsen et al., 2010a) with a constant neighborhood size equal to five and a Pearson's Correlation Coefficient significance threshold of 0.01. To distinguish between gene co-expression and possible PPI interaction complexes, GRV-N identified networks were further refined using Likelihood of Interaction (LOI) scores, implementing the method as previously described (Larsen et al., 2007) and summarized here briefly. LOI requires a large set of previously identified PPI network and a set of relevant annotations for all proteins in the set of interactions. LOI generates a table of Z-scores, indicating the likeliness or unlikeliness that two proteins will interact, based on their annotations and the frequency of proteins with those same annotations interact in a large database of known PPIs. Since there is very little information available about interacting proteins in either Laccaria or aspen, we used publically available data from The *Arabidopsis* Information Resource (http://arabidopsis.org/) for experimentally validated PPIs to build tables of LOI scores. As we consider a requirement of PPIs that physically interacting proteins must be co-localized in the cell into regions that permit physical interactions, the annotation used in LOI-score calculations was from cellular localization predictions by WoLF PSORT (Horton et al., 2007) for all predicted proteins in aspen, and *Arabidopsis*. This approach assigns the same set of sub-cellular localization annotations, using the same criteria, to every predicted protein for both the well-studied *Arabidopsis* as well as the less well characterized aspen proteome. The highest WoLF PSORT score was used to assign protein localization annotations. In the event of a tie score between highest-scoring WoLF PSORT annotations, both predicted localizations were assigned to that protein. For those proteins assigned WoLF PSORT dual localization annotations, that protein was assigned each, separate localization. For example, if a protein was predicted to localize for the WoLF annotation “nuclear:cytoplasm,” that protein was assigned both the annotations “nuclear” and “cytoplasm.” LOI scores were calculated using 10,000 random resampling iterations. The complete table of LOI scores is available in Table S2. An LOI-score >1 was considered the threshold for allowed interactions.

### Analysis of laccaria transcriptomic data

The likely mechanism by which aspen roots detect Laccaria in pre-mycorrhizal interaction is through signaling molecules, synthesized, and exported by Laccaria into the extracellular environment. Those extracellular signals are detected by aspen root and cause aspen to alter its gene expression patterns in response. While no metabolomic information was collected from the biological system, a model of the Laccaria metabolome can be generated from transcriptomic data. Predicted Relative Metabolic Turnover (PRMT) is a computational method that defines and enables exploration of metabolite-space inferred from the transcriptome (Larsen et al., 2011a). PRMT scores predict the change in turnover of metabolites (defined as the potential for consumption or production) in an environmental metabolome, given the relative abundance of genes for unique enzyme functions (UEFs) detected in different metagenomes. In this manuscript, we use the term “unique enzyme function” to describe a specific annotation applied to an enzyme, i.e., “Phosphotransferases with an alcohol group as acceptor.” We use “enzyme reactions” to refer to metabolite transformations catalyzed by an enzyme function, i.e., “ATP + D-Glycerate ↔ ADP + 3-Phospho-D-glycerate.” A UEF may catalyze more than one enzyme reaction and an enzyme reaction may be catalyzed by more than one UEF. A metabolite is a molecular compound that is a reactant or product in an enzyme reaction. In PRMT, a metabolite is never the protein product of a gene in the metagenome.

The PRMT method makes a number of assumptions. First, as with many metagenomic analyses, it assumes that relative abundance of genes for a UEF in metagenomic sequence is proportionate to relative abundance of expressed functional proteins. Second, PRMT assumes the rate of a reaction is proportionate to the amount of enzyme, and not to the concentrations of reactant or product. Finally, PRMT assumes that the metabolome can be modeled as a well-mixed reaction, disregarding compartmentalization of metabolites and activities. All UEFs annotated to a set of metagenomes are compared to reference databases of enzyme reactions to infer the set of metabolites present. A positive PRMT score indicates a greater predicted consumption of a metabolite and/or decreased synthesis. A negative PRMT score indicates a greater predicted synthesis of metabolite and/or decreased consumption.

Enzyme Commission (EC) annotations were used to define UEFs for this analysis. UEF counts were calculated as the sum of quantile-normalized RPKM expression levels of all gene models with UEF. KEGG metabolic reactions were used to identify all possible enzymatic transformations for EC annotations. The sets of allowed enzymatic transformations were limited to those present in KEGG pathways associated with aspen or Laccaria. Lists of allowed KEGG pathways are found in Table S3.

We used Pearson's Correlation Coefficient to identity correlations between predicted metabolomic turnover in Laccaria and gene expression in aspen roots. For patterns of expression in plant root, we used the average log_2_ fold change of all genes in co-expressed gene clusters. The log_2_ fold change is equal to the log_2_ for the average gene expression for a condition minus the log_2_ average expression of a cgene over all conditions. As a negative PRMT-score indicates a predicted increased capacity for a metabolites synthesis, we considered a strong negative correlation to be an indication of Laccaria metabolomic activity associated with aspen root gene expression, i.e., increased predicted synthesis of a metabolite (a negative PRMT-score) correlating with increased expression of aspen genes (a positive log_2_ fold change).

### Combine-omics methods to generate system-scale model of pre-mycorrhizal interaction

The complete set of results from gene expression analysis, protein complex prediction, transcription factor binding motif analysis, and metabolomic model prediction can be assembled into a single model of the regulatory systems of aspen pre-mycorrhizal sensing. The following rules were used to combine multi-omics models into a single model for plant-fungus mycorrhizal interaction mechanisms.

1. *Connect sensor complex to differentially regulated gene cluster:* When genes in a co-regulated gene expression cluster overlap with genes for proteins in a predicted protein sensor complex, then that sensor complex is presumed to regulate that gene cluster.
2. *Connect sensor complex to predicted Laccaria signal:* When the gene expression pattern of genes in a co-regulated aspen gene expression cluster strongly correlated by Pearson Correlation Coefficient with calculated PRMT-score for Laccaria metabolites, then that Laccaria metabolite is presumed to be a regulator of that co-regulated aspen gene cluster. Strongly correlated is defined as being in the top and bottom 0.001 percentile for all possible correlation coefficients between gene expression patterns and PRMT-scores. The mechanism of regulation of aspen genes by Laccaria metabolite is presumed to be the aspen sensor complex associated with that co-regulated gene cluster.
3. *Connect sensor complex to differentially regulated gene cluster transcription factor binding motifs:* The transcription factor binding motifs that link a sensor complex to its regulated genes is derived from the known binding motifs for transcription factors in the sensor complex and the significantly enriched binding motifs present in the co-regulated gene cluster.
4. *Connect differentially resulted gene cluster to predicted phenotype:* The specific phenotypic response of a co-regulated gene cluster is presumed to be found in the statistically significantly enriched functional annotations in that gene cluster (CBD derived *p* < 0.05).

## Results

### Transcriptomic data

Transcriptomic data, in the form of boot-strapped normalized, log_2_-tranformed RPKM values, were collected for all aspen and Laccaria biological samples. An average of 66.9% (SD 1.2%) of aspen genes were detected as significantly expressed across all samples. An average of 66.4% (SD 2.0%) of Laccaria genes were detected as significantly expressed across all samples. The gene expression values for aspen and Laccaria samples can be found in Tables S4, S5 respectively.

### Nine clusters of co-expressed genes identified in aspen root transcriptome

**Figure 4:**
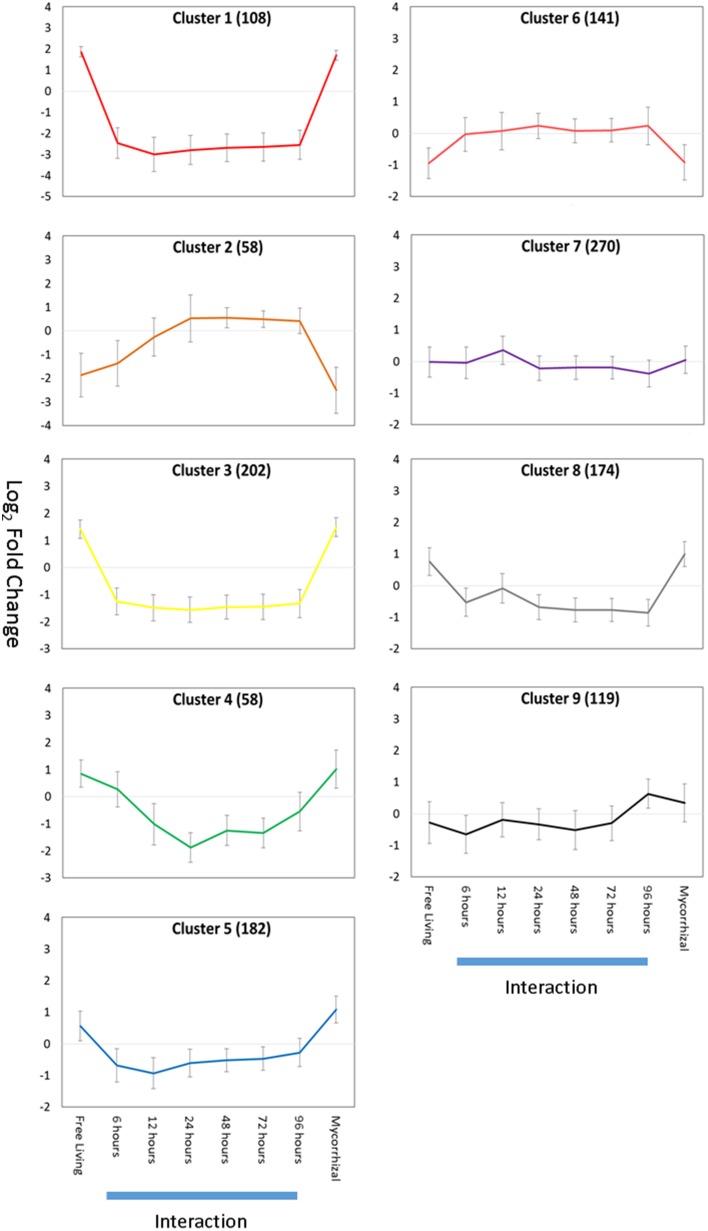
**Gene expression K-means clusters**. Aspen gene cluster co-expression for aspen was identified by K-means clustering. Y-axis is average gene expression log_2_ fold change relative to average expression over all experiments. X-axis is growth condition: FL, free living; 0–96 h, pre-mycorrhizal interaction; Myc, fully formed mycorrhizae. Error bars indicate ± one standard deviation. Blue bars below X-axis indicates gene expression data collected during root-fungus interaction samples. Numbers of gene models associated with each cluster are given in parentheses.

We examined gene expression changes at various plant-fungal interaction time points to regulatory identify regulatory changes associated with establishment of symbiosis. Aspen root gene expression was grouped into co-regulated sets of genes using K-means clustering (Figure 4). The specific genes present in each Gene Cluster are found in Table S4. Each gene cluster was found to be significantly enriched for a set of GO annotations (Table 1).

**Table 1:** **Enriched GO-biological process annotation in K-means clusters**.

**Co-expression cluster**	**Enriched GO-BP annotation**
Cluster 1	Glucan metabolism GO:0006073
	Carbohydrate metabolism GO:0005975
	Regulation of transcription, DNA-dependent GO:0006355
Cluster 2	Electron transport GO:0006118
Cluster 3	Lipid metabolism GO:0006629
	Cell wall catabolism GO:0016998
	Oligopeptide transport GO:0006857
	Carbohydrate metabolism GO:0005975
	Regulation of transcription, DNA-dependent GO:0006355
	Protein amino acid phosphorylation GO:0006468
	Electron transport GO:0006118
	Defense response GO:0006952
Cluster 4	Response to oxidative stress GO:0006979
	Transport GO:0006810
	Electron transport GO:0006118
Cluster 5	Apoptosis GO:0006915
	Defense response to pathogen GO:0042829
	Defense response GO:0006952
	Amino acid transport GO:0006865
	Aromatic compound metabolism GO:0006725
	Electron transport GO:0006118
	Protein amino acid phosphorylation GO:0006468
Cluster 6	Cell adhesion GO:0007155
	Electron transport GO:0006118
Cluster 7	Fatty acid biosynthesis GO:0006633
	Apoptosis GO:0006915
	Defense response to pathogen GO:0042829
	Defense response GO:0006952
	Protein modification GO:0006464
	Metabolism GO:0008152
	Carbohydrate metabolism GO:0005975
Cluster 8	Defense response to pathogen GO:0042829
	Defense response GO:0006952
	Apoptosis GO:0006915
	Protein amino acid phosphorylation GO:0006468
	Metabolism GO:0008152
	Regulation of transcription, DNA-dependent GO:0006355
	Electron transport GO:0006118
Cluster 9	Glucan metabolism GO:0006073
	Cell wall modification GO:0042545
	Carbohydrate metabolism GO:0005975

Specific GO biological process annotations significantly enriched (p < 0.05) in K-means clusters (from Figure 3), relative to distribution of annotations in genomic annotations.

The majority of expression clusters in aspen roots showed a gene expression change during interaction time points and a constant level of gene expression for free living and mycorrhizal samples (Down-regulated in interaction: clusters 1, 3, 4, 5, and 8. Up-regulated in interaction: clusters 2, 6, 7). Half of these gene clusters (3, 5, 7, and 9) are significantly enriched for annotations related to defense or pathogen response, suggesting that gene expression patterns differentially regulated during interaction may be associated with a general response to the presence of a fungus and not specifically associated with mycorrhizal interaction. Clusters 1 and 3 are enriched for regulation of transcription and are down-regulated during interaction samples. Only aspen root cluster 9 demonstrates an expression pattern that can be described as mycorrhizae-specific: up regulated in both interaction and mycorrhizal samples. This gene expression cluster is enriched for annotations of glucan metabolism, cell wall modification, and carbohydrate metabolism. Glucan metabolism is associated with plant-pathogen interactions (Flors et al., 2007; Rigano et al., 2007) and cell wall modification suggests changes in cell wall and cell wall permeability are required during mycorrhizal interaction.

### Eight transcription factor binding motifs drive gene expression in mycorrhizal interaction

Two methods were used for linking sensor complexes with the genes they are predicted to regulate: identifying co-regulated gene clusters that share a gene for a transcription factor present in a sensor complex and identifying statistically enriched transcription factor binding motifs present in co-regulated gene clusters. The combination of these two approaches provide complimentary information regarding possible mechanisms of gene regulation (Table 2).

**Table 2:** **Transcription factor binding motifs linked to aspen co-regulated gene clusters**.

**Co-Regulated Cluster**	**Method**	**TF Binding Motif Name**	**Sequence**	**#Genes**
Cluster 1 (106)	Enriched	MYB1AT	WAACCA	74
	Enriched	MARTBOX	TTWTWTTWTT	69
	Complex	HSE	CTNGAANNTTCNAG	1
Cluster 3 (199)	Enriched	SURE1STPAT21	AATAGAAAA	18
Cluster 5 (180)	Complex	CARGATCONSENSUS	CCWWWWWWGG	7
	Complex	E2FCONSENSUS	WTTSSCSS	28
Cluster 6 (140)	Complex	CARGATCONSENSUS	CCWWWWWWGG	3
	Complex	E2FCONSENSUS	WTTSSCSS	16
Cluster 7 (268)	Enriched	BIHD1OS	TGTCA	189
	Complex	CARGATCONSENSUS	CCWWWWWWGG	11
	Complex	E2FCONSENSUS	WTTSSCSS	26
Cluster 8 (173)	Complex	CARGATCONSENSUS	CCWWWWWWGG	11
	Complex	E2FCONSENSUS	WTTSSCSS	26
Cluster 9 (118)	Enriched	MARTBOX	TTWTWTTWTT	69
	Enriched	CAATBOX1	CAAT	118
	Enriched	CACTFTPPCA1	YACT	118
	Enriched	ARR1AT	NGATT	118
	Enriched	RBCSCONSENSUS	AATCCAA	33
	Complex	CARGATCONSENSUS	CCWWWWWWGG	7
	Complex	E2FCONSENSUS	WTTSSCSS	26

Numbers in parentheses after co-regulated cluster number is the total number of genes associated with that cluster. “Method” refers to how TF binding motif was associated with cluster: either from known TF binding motifs in regulating sensor complex, or if found to be statistically, significantly enriched in set of co-regulated genes. “#Genes” indicates the number of genes within the co-regulated cluster that have the TF binding motif at least once within 1000 bp upstream region.

#### Transcription factor binding motifs from aspen sensor protein complexes

There are four transcription factor binding motifs identified from transcription factors in predicted aspen sensor protein complexes. Heat shock element (HSE), a transcriptional activator of heat shock genes (Rieping and Schoffl, 1992; Haralampidis et al., 2002; Wenkel et al., 2006) is found in co-regulated cluster 1. E2F (E2FCONSENSUS) (Vandepoele et al., 2005) and MADS box consensus sequence (CARGATCONSENSUS) (Hepworth et al., 2002; Hong et al., 2003; Michaels et al., 2003; de Folter and Angenent, 2006) are both present in putative pathogen defense response-related aspen sensor protein complex C. Transcription factor classes IAA-AUX and Pathogen ERF elements were also present in predicted sensor protein complexes, but these transcription factor types are not associated with specific DNA binding motifs [e.g., for IAA-AUX (Ballas et al., 1993; Kim et al., 1994; Ulmasov et al., 1997; Hagen and Guilfoyle, 2002; Goda et al., 2004); and ERF (Terzaghi and Cashmore, 1995; Fujimoto et al., 2000; Koyama et al., 2003)].

#### Transcription factor binding motifs statistically enriched in co-regulated gene clusters

MYB1AT, dehydration response in Arabidopsis (Abe et al., 2003), and MARTBOX, scaffold attachment region in drosophila (Gasser et al., 1989), are transcription factor binding motifs enriched in aspen co-regulated gene clusters 1 and 3. MARTBOX suggests gene regulation by making changes to chromosomal packing. Dehydration related MYB1AT, with HSE in gene cluster 1, suggests a general stress-response regulation. SURE1STPAT21, sucrose responsive element identified in potato (Grierson et al., 1994), is enriched in cluster 3. Binding motif BIHD1OS, linked to disease resistance in rice (Luo et al., 2005), in enriched in aspen co-regulated gene cluster 7, which is also regulated by pathogen-defense associated sensor C and enriched for genes annotated as defense response. CAATBOX1, a tissue-specific promoter in pea (Shirsat et al., 1989), ARR1AT, a cytokinin-regulated transcription factor found in Arabidopsis and rice (Sakai et al., 2000; Ross et al., 2004), and CACTFTPPCA1, a regulator of phosphoenolpyruvate carboxylase of C4 dicots (Gowik et al., 2004) are all not only enriched in co-regulated gene cluster 9, but are also associated with every gene in the cluster. Additionally, DNA binding motif RBCSCONSENSUS, previously reported as light-sensitive transcription factor binding site (Manzara and Gruissem, 1988; Donald and Cashmore, 1990) is enriched in aspen co-regulated gene cluster 9.

### Five mycorrhizal-associated sensor protein complexes predicted for aspen root

**Figure 5:**
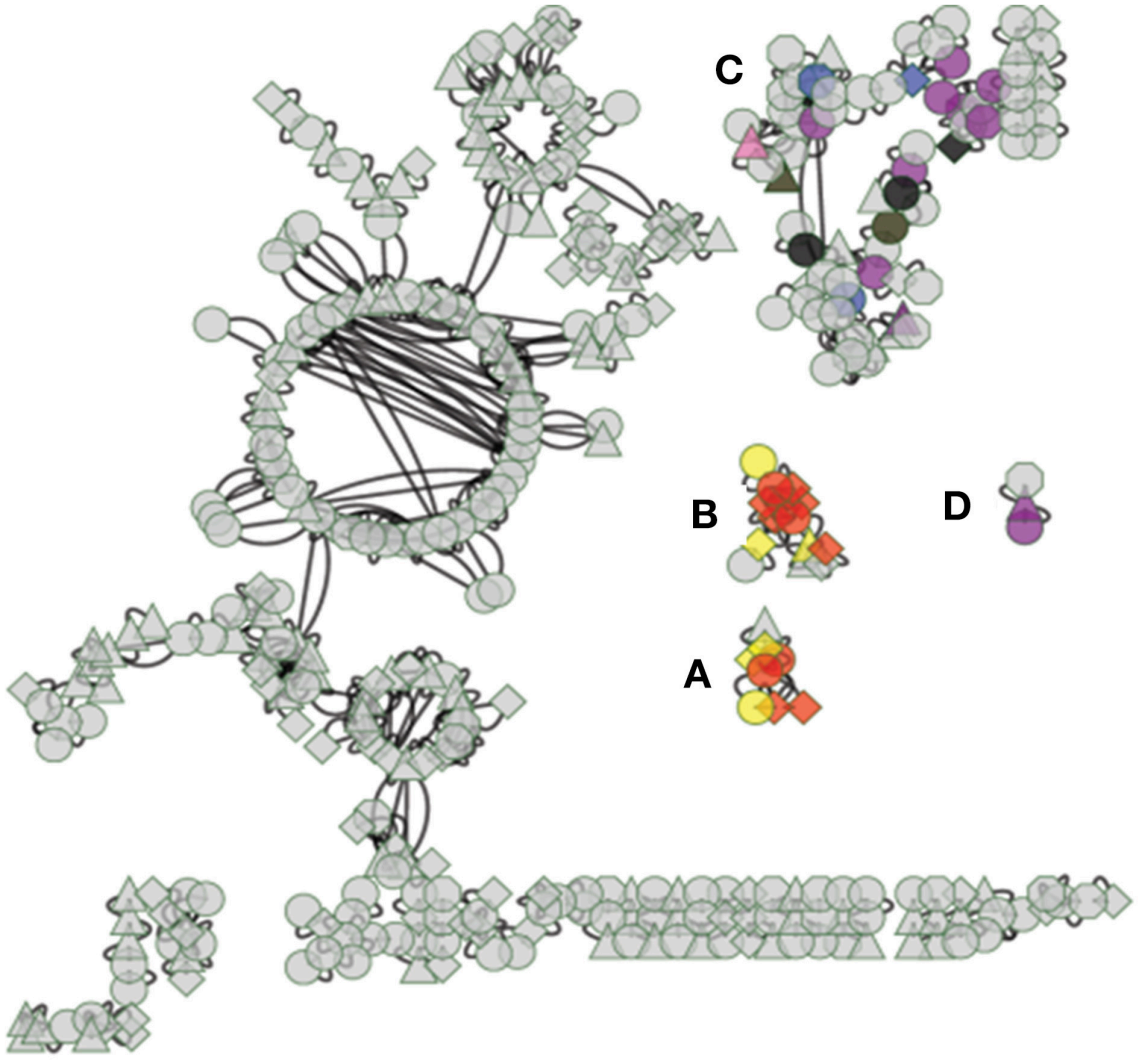
**Predicted mycorrhizal sensor complexes**. Connected subnetworks are hypothesized to be sensor complexes expressed in aspen root during mycorrhizal interaction, connecting extracellular sensory receptors to transcription factors via signal cascades. Diamonds indicate a transcription factor, triangles indicate a transmembrane receptor, circles indicate a signal cascade protein, and octagons indicate a pathogen defense response protein. Edges indicate a predicted physical interaction between expressed proteins. Nodes highlighted with color are gene models that overlap gene co-expression clusters (highlighting color uses same scheme as in Figure 3). **(A–D)** refer to predicted Protein Sensor Complexes A–D described in text. The complete set of predicted complexes is available in Table S6.

An integrated analysis of transcriptomic data, genomic annotations and previously validated plant protein-protein interactions was used to predict protein complexes in aspen root. There are 459 proteins and 536 edges in the predicted aspen PPI network (Figure 5). All predicted protein interaction pairs are available as Table S6. This network consists of a total of 77 subnetworks, of which 44 are comprised of only a pair of proteins (Larsen et al., 2011a). Four of the predicted subnetworks contain genes for proteins that overlap with co-regulated gene clusters, which are identified as complexes A–D in Figure 5. The remaining protein complexes are also predicted to be present in aspen roots, but are not predicted to be involved in the mycorrhizal sensing network and are not further considered in this analysis. Specific gene products in each predicted sensor complex A–D are found in Table S3. Each predicted sensor is described in greater detail by the annotations of its constituent proteins.

#### Aspen sensor PPI complex A

Three of the four transcription factors in this sensor complex are annotated as containing a pathogenesis-related transcriptional factor and ERF (AP2/ERF) domains (Fujimoto et al., 2000). The fourth transcription factor is annotated as a Heat shock factor (HSF)-type, DNA-binding (Clos et al., 1990).

#### Aspen sensor PPI complex B

Aspen sensor PPI complex B, contains two transmembrane receptors, one of which contains a NB ARC domain, a signaling motif found in plant resistance gene products (van der Biezen and Jones, 1998). All of the transcriptions factors in this predicted sensor complex are annotated as containing a pathogenesis-related transcriptional factor and AP2/ERF domains (Fujimoto et al., 2000).

#### Aspen sensor PPI complex C

Aspen sensor PPI complex C appears to be dedicated to pathogen defense. Twenty six percent of its members are annotated as defense response to pathogen (GO:006952). It contains two MADS-box transcription factors and one Ethylene Response Factor (E2F1) cell cycle transcription factor. The complex also contains eight genes annotated as Curculin-like (mannose-binding) lectin (IPR001480). Lectin synthesis has been observed to occur in plants in response to specific environmental stresses and lectins are involved in stress signaling (Van Damme et al., 2004; Lannoo and Van Damme, 2010; Van Dammes et al., 2011) and in plant symbiosis development (De Hoff et al., 2009).

#### Aspen sensor PPI complex D

Aspen sensor PPI complex D does not contain any transcription factors. It does contain a receptor disease resistance protein often correlated with a hypersensitive response and previously reported to confer resistance to bacterial, viral, and fungal pathogens (Staskawicz et al., 1995).

### Laccaria predicted to signal aspen roots with plant hormones, terpenoids, and phenylpropanoids

We used the PRMT approach (Larsen et al., 2011a) to derive metabolic models from the *Laccaria* transcriptome and identify KEGG pathways with possible involvement in mediating the establishment of plant symbiosis with the plant. The complete Laccaria metabolic network contains 1269 enzyme-mediated reactions between 904 metabolites and 387 UEFs (Table S7 contains the metabolic network and Table S8 contains all calculated PRMT scores). The largest connected subnetwork consists of 458 metabolites and 881 reactions. A total of 39 Laccaria PRMT scores were found to have strong correlations with observed patterns of aspen root gene expression (Table 3). A strong negative correlation indicates that when a compound is predicted to be synthesized by *Laccaria*, there is a corresponding change in the expression of genes in the aspen root transcriptome. KEGG pathways indicated to be involved in synthesis of signaling compounds are Biosynthesis of Plant Hormones (KEGG map07070), N-Glycan Biosynthesis (map00510), Biosynthesis of Alkaloids Derived from Shikimate Pathway (map01063), Biosynthesis of Phenylpropanoids (map01061), and Biosynthesis of Plant Secondary Metabolites (map01060). These identified KEGG pathways correlate well with previously published observations of mycorrhizal interaction. The Biosynthesis of Plant Hormones (KEGG map07070) pathways include those for auxin, ethylene, jasmonic acid, and brassinosteroids which have all been previously reported as signaling compounds in plant-symbiote interactions (Sun et al., 2006; Glick et al., 2007; Sukumar et al., 2013). In addition, terpenoids have been linked to interactions with plant roots and root morphology (Umehara et al., 2008). N-linked glycans (N-Glycan Biosynthesis, map00510) play an important role in cell-cell interaction (Van Damme et al., 2004; Lannoo and Van Damme, 2010; Van Dammes et al., 2011) and N-linked glycans are potentially associated with the sugar-binding lectins in sensor protein complex A. The Biosynthesis of Phenylpropanoids (map01061) includes compounds such as catechin, epicatechin, and 4-hydroxybenzoate 4-OP-glucoside which have been identified as crucial in plant-fungal interactions, particularly for early mycorrhization (Campbell and Ellis, 1992; Weiss et al., 1997). Salicylate, which correlates with aspen gene co-expression cluster 1, is a plant hormone modulating inducible plant defenses (Feys and Parker, 2000; Thaler et al., 2012).

**Table 3:** **Predicted Laccaria metabolism correlates with clustered aspen root gene expression**.

**Correlates with Aspen Gene Cluster**	**Laccaria Metabolite**	**Relevant KEGG Pathway**
Cluster 1	Caffeoyl-CoA	**Biosynthesis of phenylpropanoids**
	Salicylate	**Biosynthesis of plant hormones**
	Pyruvate	
	trans-2,3-Dihydroxycinnamate	**Degradation of aromatic compounds**
	3-(2,3-Dihydroxyphenyl) propanoate	
	Hydantoin-5-propionate	Histidine metabolism
	Dolichyl beta-D-glucosyle phosphate	**N-Glycan biosynthesis**
	Dolichyl diphosphate	
	N-Hydroxyphenylacetate	Phenylalanine metabolism
	Dextrin	Starch and sucrose metabolism
	N-Hydroxyl-tryptamine	
	2-Octaprenyl-3-methyl-5-hydroxy-6-methoxy-1,4-benzoquinone	**Ubiquinone and other terpenoid-quinone biosynthesis**
	Aldoxime	
	4-Hydroxymandelonitile	
Cluster 2	Pyruvate	**Biosynthesis of plant hormones**
	1-Phosphatidyl-D-myo-inositol	**N-Glycan biosyntesis**
	Dolichyl beta-D-glucosyle phosphate	
	Dextrin	Starch and sucrose biosynthesis
Cluster 5	L-Gilonon-1,4-lactone	Ascorabate and aldarate metabolism
	beta-D-Fructose 1,4-bisphosphate	**Biosynthesis of plant secondary metabolites**
	Pyruvate	
	Ocanoyl-CoA	
	Lauroyl-CoA	
	Tetradecanol-CoA	Fatty acid metabolism
	Decanoyl-CoA	
	Hexanoyl-CoA	
Cluster 6	Benzoate	**Biosynthesis of alkaloids derived from shikimate pathway**
	p-Coumaroyl-CoA	**Biosynthesis of phenylpropanoids**
	L-Asparagine	**Biosynthesis of plant secondary metabolites**
	Tryptamine	
	(Z)-4-Hydroxyphenylacetaldehyde-oxime	Glucosinolate biosynthesis
	4-Imidazolone-5-propanoate	Histidine metaolism
	Nitrile	Nitrogen metabolism
	Fe^2+^	
	3-Coumaric acid	Phenylalanine metabolism
	3-(3-Hydroxyphenyl)-propanoic acid	**Ubiquinone and other terpenoid-quinone biosynthesis**
	2-Hexaprenyl-6-methoxyphenol	
	2-Ocatprenyl-3-methyl-6-methoxy-1,4-benzoquinone	

To identify potential signaling molecules and classes of signaling molecules synthesized by Laccaria and detected by aspen root, predicted differentially metabolized Laccaria molecules were correlated with observed patterns of aspen root gene expression. Column “Aspen Gene Cluster” identifies the aspen co-expressed gene clusters from Figure 4. “Laccaria Metabolite” lists the predicted Laccaria metabolites with PRMT-scores that strongly, negatively correlate (0.005/0.5th percentile of all correlations between gene expression patterns and PRMT-scores) with aspen root gene co-expression patterns. While a metabolite might belong to multiple KEGG pathways (Ogata et al., 1999), the KEGG pathway predicted to be most relevant to Laccaria-aspen signaling compounds is identified for each metabolite. Specific KEGG pathways likely to be relevant to mycorrhizal signaling are highlighted in bold.

Lipid are observed to correlate with aspen gene co-expression cluster 5. Although lipids may not be good candidates for diffusible signals in this experimental design due to the unlikelihood that lipids can diffuse through the permeable membrane, lipids can be signal molecules in that regulate developmental processes, and response to pathogens (Howe and Jander, 2008; Brodhun and Feussner, 2011). Also, Fatty acid biosynthetic pathways, particularly Hexanoyl-CoA, are precursors for jasmonate biosynthesis (KEGG map01070) and jasmonic acid is a previously reported important mycorrhizal signaling compound (Bari and Jones, 2009; Gutjahr and Paszkowski, 2009).

Some strongly correlating metabolites are unlikely to be directly involved in signaling, but may indicate corresponding metabolic activities in Laccaria interacting with plant root. These metabolites include pyruvate and dextrin, which are non-specific to any particular metabolic process but do occur in pathways such as Biosynthesis of plant secondary metabolites and Biosynthesis of plant hormones and may indicate general shifts in metabolic and energy use priorities by pre-mycorrhizal Laccaria.

### A model of fungus-plant signaling and regulation in mycorrhizal interaction

**Figure 6:**
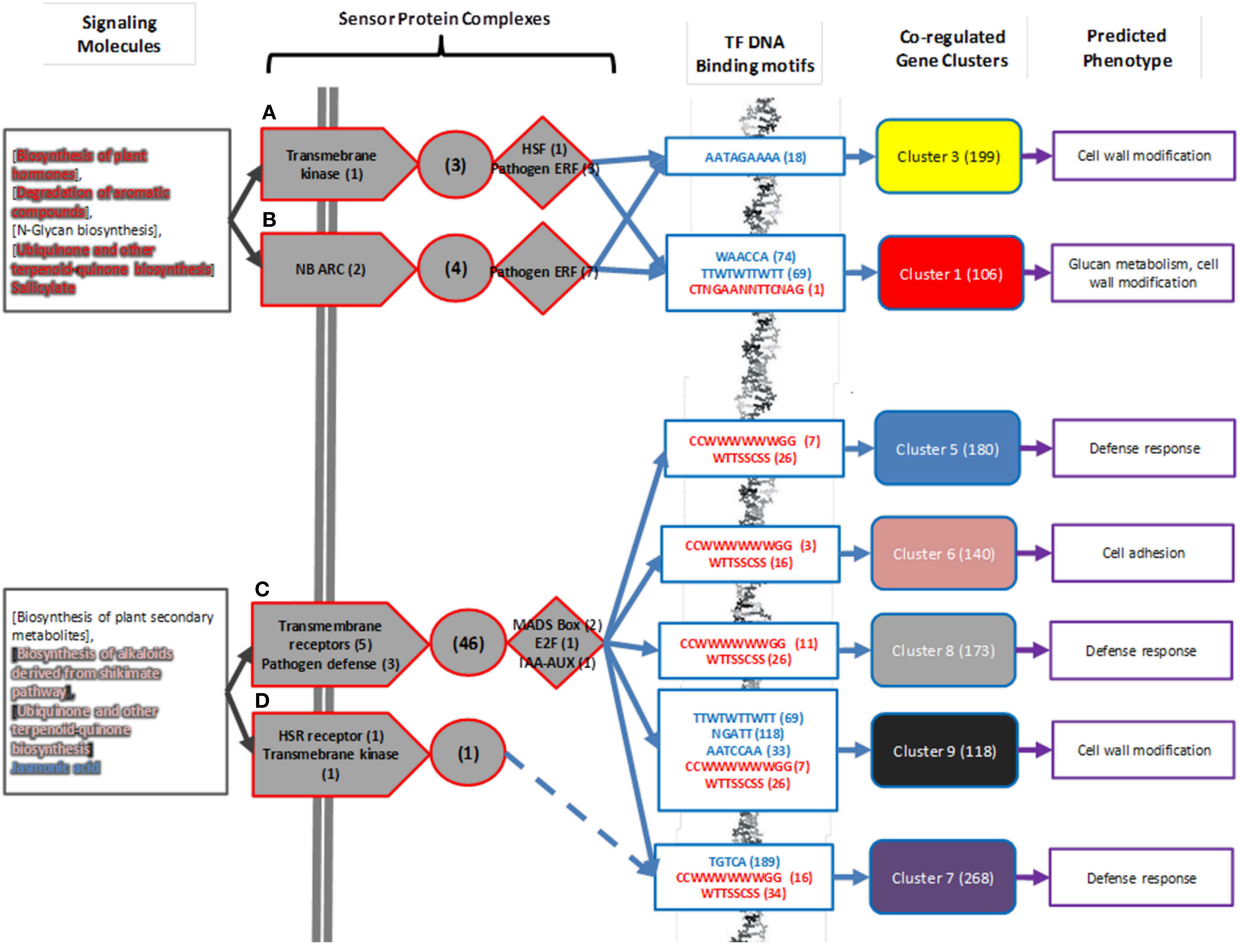
**Summary for predicted regulatory mechanisms of mycorrhizal sensing**. Predicted classes of signaling molecules (Gray boxes) are collated from Table 3. In this figure, as in Figure 4, transmembrane receptors are arrows, signal cascades are circles, and transcription factors are diamonds. Specific genes in predicted sensor complexes (from Figure 4) are summarized by protein function. **(A–D)** refer to predicted Protein Sensor Complexes A–D described in text. Numbers in parentheses are number of proteins associated with function present in predicted sensor complex. DNA binding motifs associated with sensor complex are identified by known motifs from transcription factors in sensor complexes (highlighted red) or are identified as statistically significantly enriched in co-regulated gene clusters (highlighted blue). Numbers in parenthesis indicate number of gene models in co-regulated gene cluster that have transcription factor DNA binding motif in 1000 bp upstream region. Co-regulated gene expression clusters are highlighted using same scheme as in Figure 3. Predicted phenotype as a consequence of gene regulation is summarized from Table 1. Numbers in parentheses indicate number of gene models associated with co-regulated gene cluster.

The complete set of results from gene expression analysis, protein complex prediction, transcription factor binding motif analysis, and metabolomic model prediction can be assembled into a single model of the regulatory circuitry of aspen mycorrhizal sensing (Figure 6).

All of the predicted sensor complexes contain pathogen or fungal-response related receptors or transcription factors, indicating that they are relevant to interaction with the fungus Laccaria. The predicted mycorrhizal sensor network is divided into two main components. The first component is comprised of sensor complexes A and B are predicted to detect N-glycans and aromatics and control modification to aspen root cell walls. There are three transcription factor binding motifs unique to this regulatory mechanism. The second component, made up of Sensor complexes C and D, detect jasmonic acid and other plant-like metabolites synthesized by Laccaria and drives root response to pathogens. Sensor complex D does not include a transcription factor, but may modulate gene expression of defense response gene cluster 7 by interaction with complex C through its defense response to pathogen associated protein kinase. There are seven transcription factor binding motifs unique to this regulatory mechanism.

**Figure 7:**
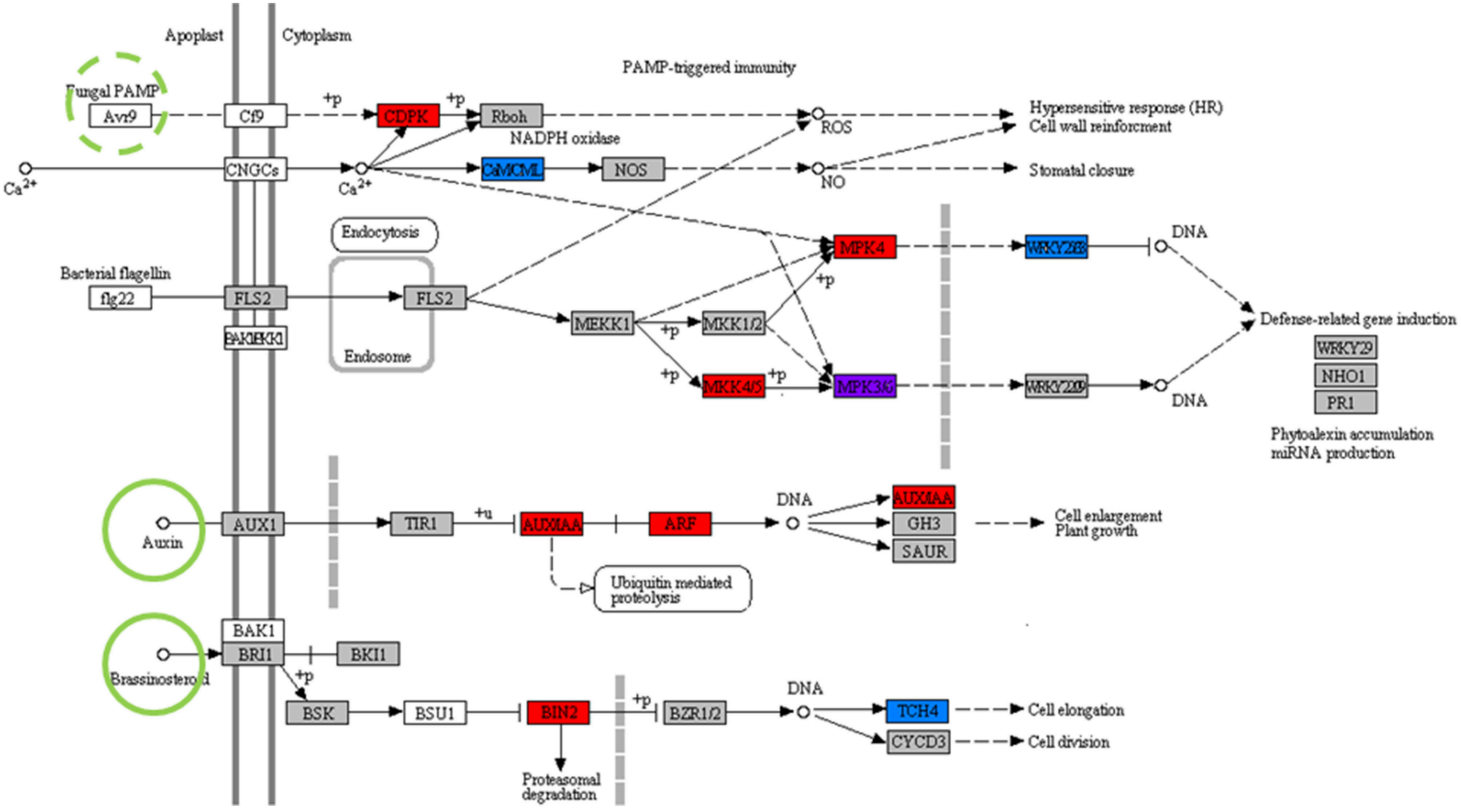
**Predicted symbiote recognition pathways overlap with prior knowledge**. The network combines portions of regulatory cascades from KEGG pathways for Plant-pathogen interactions (Map 04626) and Plant hormone signal transduction (Map 04075). All highlighted nodes have homologs in *P. trichocapra*. **Red** highlighted nodes are present in aspen sensor complexes, **blue** highlighted nodes are present in aspen co-regulated clusters, and the **purple** highlighted node is present in both regulatory clusters and sensor complexes. **Green** highlighted ligands overlap predicted Laccaria metabolism. Fungal PAMP (Pathogen Associated Molecular Patterns), highlighted by green dashed-circle, refers to a broad class of ligands and is not directly predicted by metabolic model but can be inferred to be present in Laccaria-aspen interaction system.

In addition to the support for individual components of the model prediction in the available literature as described in previous sections, the system-scale network in Figure 6 is also supported by overlap with previously reported plant interaction sensor mechanisms. Predicted regulator circuitry intersects with known KEGG pathways for Plant-Pathogen Interactions (map04626) and Plant Hormone Signal Transduction (map04075) (Figure 7). Of the 30 proteins in this combined symbiote recognition pathway, 11 are coded for by genes in the predicted interaction network. Of particular interest from this overlap is the signal pathway connecting Laccaria-synthesized plant hormones auxin and brassinosteroids to cell elongation. Auxin synthesized by Laccaria has been previously reported as driving root elongation phenotype (Sukumar et al., 2013). Also, the fungal PAMP (pathogen-associated molecular patterns) signal cascade is well represented in our predicted regulatory model. Specifically, the signal pathway for down-regulating defense-related gene induction is represented in our predicted network, which we propose is the signaling pathway by which Laccaria inhibits aspen root's fungal pathogen response in order to establish mycorrhizal interaction. While there is no known aspen homolog for fungal PAMP receptor in the curated regulatory network, we propose that the PAMP receptor is one of the 10 transmembrane receptors in predicted sensor complex C or D (Figure 3).

## Discussion

The complete set of results from gene expression analysis, protein complex prediction, transcription factor binding motif analysis, and metabolomic model prediction can be assembled into a single model of the regulatory circuitry of aspen mycorrhizal sensing. The model predicts the class of signaling compounds of terpenoids and the specific signaling molecules jasmonic acid and salicylate are diffusible signals synthesized by Laccaria during mycorrhizal interaction. There are four predicted aspen root sensor protein complexes that detect these diffusible signals through 13 transmembrane receptors and regulate the activity of 15 transcription factors. The transcription factors are predicted to interact with 8 possible transcription factor DNA binding motifs on the aspen genome to regulate the expression of 1184 genes. The phenotype controlled by these genes is predicted to be the modulation of defense response to pathogenic fungus, the modification of root cell walls, and the alteration to root structure morphology.

Not every possible interaction implied by these results is explicitly identified in this model, but there are secondary regulatory mechanisms that can be inferred. For example, the HSF transcription factor in aspen sensor protein complex A is predicted, by analysis of DNA binding motifs, to regulate another transcription factor (aspen JGI gene model 830963) present in regulated gene cluster 3. This transcription factor is annotated as DNA-binding WRKY (DNA binding motif, TTTGACY) (Rushton et al., 1996; Eulgem et al., 2000). There are 33 gene models in co-regulated clusters 3 and 16 and in cluster 1 that have the WRKY DNA-binding motif within the 1000 bp upstream of their coding sequences (16% of genes in both co-regulated clusters, predicted to be regulated by sensor protein complex A). DNA-binding WRKY regulation is associated with plant developmental programs, including pathogen defense (Eulgem et al., 2000). This suggests that there may actually be two levels of interaction present in this system: HSF transcription factor regulates the transcription factor 830963 which in turn, regulates additional cell wall modification genes in response to pathogens.

These predictions identify specific biological experiments to validate the models, although performing even a small subset of the possible experiments proposed by these results is beyond the scope of this manuscript. For example, treating aspen roots with predicted signaling compounds should produce a characteristic gene expression pattern that is a predictable subset of the gene expression changes observed in the interaction experiments. Predicted transmembrane receptor proteins can be synthesized and purified to validate their predicted binding ligands *in vitro*. ChIPseq experiments using the predicted mycorrhizal sensing mechanism transcription factors can validate predicted gene regulatory mechanisms. We anticipate that the generated model will yield ample opportunity for additional biological experimentation. The computational modeling approach used here also can be generalized to explore additional mechanisms of symbiotic interactions, provided that there is a similar body of prior observations from which protein interaction networks and gene expression patterns over a wide range of conditions are available.

## Author contributions

Conceived and designed the experiments: FC, LC, PL. Performed the experiments: AS, GT. Analyzed the data: FC, LC, PL, YD, SD. Contributed reagents/ materials/analysis tools: LC, YD, PL. Wrote the paper: FC, LC, PL All authors read and approved the final manuscript.

## Funding

This contribution originates in part from the “Environment Sensing and Response” Scientific Focus Area (SFA) program at Argonne National Laboratory. The submitted manuscript has been created by UChicago Argonne, LLC, Operator of Argonne National Laboratory (“Argonne”). Argonne, a U.S. Department of Energy Office of Science laboratory, is operated under Contract No. DE-AC02-06CH11357. The U.S. Government retains for itself, and others acting on its behalf, a paid-up nonexclusive, irrevocable worldwide license in said article to reproduce, prepare derivative works, distribute copies to the public, and perform publicly and display publicly, by or on behalf of the Government.

### Conflict of interest statement

The authors declare that the research was conducted in the absence of any commercial or financial relationships that could be construed as a potential conflict of interest.

## Availability of supporting data

Expression data, all metabolic and PPI networks, and PRMTscores are available as Supplemental Data. Raw sequence data is available upon request.

## Supplementary material

Table S1 **A list of allowed GO annotations used for proteins in aspen sensor networks**.

Table S2 **LOI score table used in prediction of aspen sensor complex PPIs**.

Table S3 **A List of allowed KEGG pathways used in Laccaria metabolome model**.

Table S4 **Aspen gene expression data, as log_2_ RPKM, calculated using BowStrap**. For every biological sample, there are three columns of data derived from BowStrap analysis: Average RPKM, PRKM Standard Deviation, and a p-value indicating significance of detected gene expression. Two additional data columns indicate Gene Cluster membership (Clusters 1–7 as identified in Figure 3. “0” indicates that the gene does not belong to any cluster) and Sensor Complex membership (Complexes A–D as identified in Figure 4. “none” indicates that the gene product is not a member of any predicted complex).

Table S5 **Laccaria gene expression data, as log_2_ RPKM, calculated using BowStrap**. For every biological sample, there are three columns of data derived from BowStrap analysis: Average RPKM, PRKM Standard Deviation, and a p-value indicating significance of detected gene expression.

Table S6 **Predicted aspen sensor complex PPI networks as a list of protein-protein interaction pairs**.

Table S7 **Laccaria metabolome network as a list of interactions in the format: reactant, enzyme activity, product**.

Table S8 **Table of all Laccaria metabolome PRMT scores**.

## Abbreviations

CBD: Cumulative Binomial Distribution
EC: Enzyme Commission
ECM: Ectomycorrhizal
GO: Gene Ontology
JGI: Joint Genome Institute
KEGG: Kyoto Encyclopedia of Genes and Genomes
MMN: Modified Melin Norkan's
RPKM: Reads Per Killobase: per Million RNAseq reads
WPM: Woody Plant Media.
